# Changes in Students’ Understanding of and Visual Attention on Digitally Represented Graphs Across Two Domains in Higher Education: A Postreplication Study

**DOI:** 10.3389/fpsyg.2020.02090

**Published:** 2020-08-27

**Authors:** Sebastian Brückner, Olga Zlatkin-Troitschanskaia, Stefan Küchemann, Pascal Klein, Jochen Kuhn

**Affiliations:** ^1^Chair of Business and Economics Education, Johannes Gutenberg-University Mainz, Mainz, Germany; ^2^Physics Education Research Group, Technische Universität Kaiserslautern, Kaiserslautern, Germany; ^3^Faculty of Physics, Georg August University Göttingen, Göttingen, Germany

**Keywords:** graph understanding, pretest–posttest, eye-tracking, dwell times, confidence rating, university students

## Abstract

Domain-specific understanding of digitally represented graphs is necessary for successful learning within and across domains in higher education. Two recent studies conducted a cross-sectional analysis of graph understanding in different contexts (physics and finance), task concepts, and question types among students of physics, psychology, and economics. However, neither changes in graph processing nor changes in test scores over the course of one semester have been sufficiently researched so far. This eye-tracking replication study with a pretest–posttest design examines and contrasts changes in physics and economics students’ understanding of linear physics and finance graphs. It analyzes the relations between changes in students’ gaze behavior regarding relevant graph areas, scores, and self-reported task-related confidence. The results indicate domain-specific, context- and concept-related differences in the development of graph understanding over the first semester, as well as its successful transferability across the different contexts and concepts. Specifically, we discovered a tendency of physics students to develop a task-independent overconfidence in the graph understanding during the first semester.

## Research Focus and Objective

The ability to understand digitally represented graphs is a necessary prerequisite for (online) learning in most disciplines in higher education^1^ (Bowen and Roth, 1998). In general, graphs are used to simplify the presentation of (complex) concepts and to facilitate the exchange of information between individuals (Curcio, 1987; Pinker, 1990; Freedman and Shah, 2002). Because the graphical representation of information is increasingly becoming more important than texts in the online information landscape (Moghavvemi et al., 2018), the ability to interpret graphs is considered a central facet of cross-domain generic skills such as online reasoning (Wineburg et al., 2018), media literacy (Shah and Hoeffner, 2002), data literacy (Cowie and Cooper, 2017), and information problem-solving (Brand-Gruwel et al., 2009).

Because graphs and other types of diagrams are an instructional method for representing both domain-specific and generic knowledge, they are the main focus in teaching, especially at the beginning of university studies (Heublein, 2014; Laging and Voßkamp, 2017). Usually, the graphs are embedded in text-based instructions to aid the comprehension of textual descriptions and to supplement these descriptions by providing the learner with further visually structured information (Stern et al., 2003).

Line graphs, in particular, are frequently used in higher education. For example, the relationships between distance and speed in physics or between time and stock prices in finance can both be illustrated with a line graph (Bowen and Roth, 1998; Benedict and Hoag, 2012; Susac et al., 2018; Becker et al., 2020a, b; Hochberg et al., 2020; Klein et al., 2020). More recently, a number of studies investigated and compared university students’ understanding of graphs in mathematics, physics, and other contexts using parallel (isomorphic) tasks (Christensen and Thompson, 2012; Planinic et al., 2012, 2013; Wemyss and van Kampen, 2013; Bollen et al., 2016; Ivanjek et al., 2016, 2017). These studies have shown that parallel tasks with an added context (physics or other context) were more difficult to solve than the corresponding mathematics problems and that students who successfully solve problems in (purely) mathematical contexts often fail to solve corresponding problems in physics or other contexts. Other studies have discovered that students often struggle to interpret line graphs or solve problems based on line graphs (Canham and Hegarty, 2010; Kragten et al., 2015; Miller et al., 2016). Students do not succeed in transforming data into line graphs (Bowen and Roth, 1998); they do not spend sufficient time trying to understand the depicted concepts (Miller et al., 2016) or have difficulties comprehending the underlying concept.

Although the major importance of being able to correctly interpret visual representations and graphs within and across domains (Beichner, 1994; Stern et al., 2003; Planinic et al., 2013), which must be distinguished from the ability to understand textual representations (Mayer, 2009), is widely known and recognized, research on the ability of students in higher education to solve problems with digitally represented graphs combined with results on how students extract information from graphs within and across domains is still scarce. In particular, there are only very few studies on the development of students’ graph understanding over a degree course.

In this paper, we address this research deficit in a post-replication study by following up on two existing studies by Susac et al. (2018) and of Klein et al. (2019). Both studies investigated students’ allocation of visual attention, i.e., how students extract information from graphs, during problem-solving in relation to their scores. In our study, we extend this approach by including a comparison of pre- and post-test results. For this purpose, we use the same graph tasks from the two domains (physics and economics) that were chosen in the two reference studies. To gain initial insights about a change in students’ graph comprehension within and across domains, we also retest a subset of the same students who previously participated in Klein et al.’s study (2019) at the end of their first semester.

To achieve a higher degree of (external) validity and generalizability, the replication of a study requires a comprehensive presentation of the control variables and can expand the original study in some aspects (Schmidt, 2009). The study presented here, in addition to a replicating previous research, was expanded through the addition of the second measurement point. As learning with graph tasks, especially in first semester lectures, is an integral part of the curriculum and instruction in both domains examined here (e.g., Jensen, 2011), more in-depth knowledge and skills can be acquired by attending such lectures, and a change in graph understanding in these two domains can be expected. Thus, in this post-replication study, changes in students’ understanding of graphs are investigated within and across the two domains physics and economics. Moreover, previous research indicates that while students’ understanding of graphs can improve after a targeted intervention, students did not improve in transferring this ability to different task contexts (e.g., Klein et al., 2015). Therefore, in this study, we investigate whether eye movements are indicative of increases in graph understanding and potential weaknesses in transferring graph understanding across different domains and contexts.

Based on these studies, we developed the following research questions (RQ) for this article, which focus on the theoretically expected (i) time effects (measurements t1 and t2), (ii) domain effects (physics and economics), (iii) (task) context effects, and possible (vi) interaction effects:

• RQ1: To what extent does the ability of students from both domains to solve line-graph problems in physics and finance contexts change over the course of the first semester?
• RQ2: Are the confidence ratings of graph task solutions in physics and finance contexts of students from both domains higher at the end of the semester, and how do they change with respect to correct and incorrect responses?
• RQ3: How do the dwell times on specific parts of graph tasks in physics and finance contexts of students from both domains change between the beginning and the end of the semester?

In the following, the two studies by Susac et al. (2018) and Klein et al. (2019) that this replication study is based on are described in detail. Next, we expand the focus on the two domains examined and theoretically ground the additional research focus on the development of the students’ graph understanding. The hypotheses for this study are formulated based on the defined conceptual and methodological frameworks. These, in turn, are based on the method of eye-tracking (ET).

## Background of the Post-Replication Study

### Cross-Sectional Studies by Susac et al. (2018) and Klein et al. (2019)

In a recent study in Croatia (Susac et al., 2018) and a German replication study (Klein et al., 2019), students’ graph understanding in physical and economic tasks was experimentally investigated for the first time. In a 2 × 2 × 2 study design, permuted systematically according to three characteristics, the graphical concept (graph “slope” vs. “area” under the curve), the type of question (quantitative vs. qualitative), and the domain-specific context (physics vs. finance) were differentiated into four isomorphic task pairs (eight tasks in total; Klein et al., 2019; Susac et al., 2018). Comparing the students from two different domains (in Susac et al., students of physics and psychology responding to graph tasks from physics and finance; in Klein et al., students of physics and economics responding to graph tasks from physics and finance), both studies confirm the differences between the two domains; for instance, physics students spend a longer period of time on unknown axis tick labels and analyzing the curve, whereas within the domain of economics, students there were no significant differences (Susac et al., 2018; Klein et al., 2019).

In the cross-sectional study by Susac et al. (2018), students often found it more difficult to make calculations based on the graph concept “area” (e.g., using an integral) than to determine the “slope” of a graph. This result confirms existing findings and theoretical assumptions (Cohn et al., 2001; Benedict and Hoag, 2012). Klein et al. (2019) found that “area” tasks required more time and were therefore cognitively more demanding than “slope” tasks for both domains (physics and economics).

With regard to the transfer of task solutions across domains, both studies found that physics students, who are the better task solvers in one task context (physics), also performed better in another context (finance). For instance, physics students achieved similarly good results in graph understanding in both examined domains, however, they solved the tasks from the domain of physics more quickly than the tasks from the domain of economics. Psychology students generally scored comparatively lower in graph understanding (Susac et al., 2018). Klein et al. (2019) found similar differences.

Comparing tasks that require calculations (quantitative) and those that require only verbal interpretation (qualitative), both studies demonstrated that quantitative tasks are generally more challenging as the students achieved lower scores and at the same time took longer to complete these tasks (Susac et al., 2018; Klein et al., 2019). This finding is in line with existing research, indicating that students have specific difficulties when solving tasks with numerical or mathematical requirements (Planinic et al., 2012; Shavelson et al., 2019).

In addition to an analysis of task scores and retention times, Klein et al. (2019) also collected the students’ self-assessments of their task solutions and compared them with the actual scores. The metacognitive assessment provided further significant insights into the students’ expertise, in particular between high- and low-performing students. In line with this existing research, Klein et al. (2019) found better self-assessments among high-performing students (Brückner and Zlatkin-Troitschanskaia, 2018) and a systematic overestimation of their own abilities among low-performing students (Kruger and Dunning, 1999). The physics students provided correct answers with higher confidence ratings in comparison to instances when they gave incorrect answers, whereas economics students who achieved lower scores also gave lower confidence ratings with regard to their own performance.

For the postreplication study, the following assumptions can be summarized:

– There are significant differences between students from the two domains when it comes to solving graph problems from familiar versus unfamiliar contexts.
– Students with high test scores assess the correctness of their solutions more precisely than students with lower test scores.
– Graph tasks with a focus on the “area under the curve” or with quantitative requirements are more difficult for students from both domains than tasks on the concept of “slope” or without mathematical requirements. This applies to both task contexts (physics and finance).

Because the data of the study by Susac et al. (2018) and the replication study (Klein et al., 2019) only originated from assessment at one point in time, changes that must be expected over the course of a semester cannot be described. As longitudinal studies indicate a significant change in knowledge during the first semester (Happ et al., 2016; Chen et al., 2020; Schlax et al., 2020), our postreplication study was expanded to include a so far underresearched developmental focus.

### Development of Graph Understanding

Through the systematic use of learning tasks comprising graph representations in different domains, especially at the beginning of studies, a positive development of graph understanding can be assumed because the acquisition of domain-specific knowledge is expected to support students in solving typical domain-specific problems related to graphs (e.g., McDermott et al., 1987). However, there are currently only few studies with a pretest–posttest assessment design focusing on the changes in graph understanding and how to foster this understanding. Digital learning environments, learning from examples, and using instructional material showed an impact on students’ graph comprehension (Bell and Janvier, 1981; Bergey et al., 2015; Becker et al., 2020a, b; Hochberg et al., 2020). For example, the impact of instruction on graph construction conventions (e.g., on legends and labels) on students’ graph understanding was confirmed in a control group design (Miller et al., 2016). By systematically training the (prospective) teachers as well as the students over several weeks, graph understanding in biology was promoted (Cromley et al., 2013).

In supplementing the instruction of graph use with material for understanding multiple representations (e.g., how data can be visualized in a graph or how information for graph use can be meaningfully extracted from a text), multiple ways to promote graph understanding over a period of 2 months were identified (Bergey et al., 2015). In a study with an augmented reality intervention over a period of 3 weeks, students of the intervention group showed an improvement in understanding that exceeded the increase in understanding of a control group with no such intervention (Jerry and Aaron, 2010). In some studies, a positive relationship between textual perception and the understanding of visual representations of domain-related concepts was found only among students with poor spatial abilities (e.g., Bartholomé and Bromme, 2009). Overall, a positive, instructionally initiated development of graph comprehension was found for several educational levels, types of instruction, and domains (Miller et al., 2016).

The development of graph understanding is often considered a generic skill that is also transferable to graphs in other contexts and domains (Miller et al., 2016). So far, however, recent research indicates learner difficulties in transferring graph understanding across problems and domains (Bergey et al., 2015; Cromley et al., 2013). For instance, one pretest–posttest study investigated to what extent university students succeed in applying mathematical functions for the “slope” of a curve to the context of physics (Woolnough, 2000). The posttest after 1 year showed an improved calculation and interpretation of the gradient, as well as a frequent use of the concept of proportionality, but the students had difficulties with the transfer from model to real world. Bergey et al. (2015) and Cromley et al. (2013) showed that training based on graph uses and learning with multiple representations can improve the understanding of graphs in biology, but the students did not succeed in transferring their skills to graphs in geoscience.

Although the ability to understand graphs is necessary for the development of domain-specific knowledge and conceptual change, especially in the domains of physics and economics^2^, so far little research has been conducted on the development of graph understanding in these two domains. Whereas in physics, and especially in physics education, there are many studies on graph understanding in kinematics (McDermott et al., 1987; Beichner, 1994; Planinic et al., 2013; Wemyss and van Kampen, 2013; Klein et al., 2020), in economics, no research field has yet been established that explicitly analyzes graph understanding (Cohn et al., 2001; Stern et al., 2003; Benedict and Hoag, 2012). In particular, it is yet underresearched to what extent a transfer between more distant disciplines, such as between natural and social science disciplines, can succeed.

While Klein et al. (2019) showed the connection between the self-assessment of solutions and the correct answers to these graph tasks, there are hardly any studies that investigate this relationship over time. However, prior, longitudinal research has identified correlations of this kind in studies using general knowledge tests (without graphs; Cordova et al., 2014; Brückner and Zlatkin-Troitschanskaia, 2018). Moreover, the Dunning–Kruger effect (Kruger and Dunning, 1999) suggests that learners with a low level of knowledge struggle to rate their own performance accurately in self-assessments. Based on these results, it can be assumed that an increase in (conceptual) knowledge and (graph) understanding is accompanied by a more precise self-assessment of knowledge.

In summary, based on research questions 1 and 2, the following hypotheses (H) can be formulated and will be examined in this study with regard to the changes in graph understanding:

• H1: Physics and economics students solve graph tasks related to the subject they are enrolled in more successfully at the second measurement point than at the first measurement point.
• H2: Physics and economics students rate their confidence in their solution of tasks related to the subject they are enrolled in more accurately at the second measurement point than at the first measurement point.

### Eye-Tracking and Graph Understanding

In recent years, ET is increasingly used to study visual representations in general (e.g., Küchemann et al., 2020b) and graph understanding in particular (Madsen et al., 2012; Planinic et al., 2013; Klein et al., 2017, 2020) as it offers many advantages, especially for uncovering the systematics underlying the perception of different graphical representations (Küchemann et al., 2020a) and can also supplement the findings on changes in test scores and self-assessments with evidence obtained from changes in eye movements. This method is also used in the two studies by Susac et al. (2018) and Klein et al. (2019) referenced here.

According to the Eye-Mind-Hypothesis (Just and Carpenter, 1980), there is a strong spatiotemporal and causal connection between visual attention and the associated cognitive processes. The visual representation of graphs includes, for instance, axes and labels, which can be arranged in different ways and, depending on the intensity and duration of the observation, can also impact understanding of the graph. For example, a comparatively longer fixation time on relevant areas of a graph was mainly observed in students who solved a task correctly (Madsen et al., 2012; Susac et al., 2018; Klein et al., 2019). Regarding the dwell time for processing one task, students’ previous experience and familiarity with tasks of this kind ease their comprehension; thus, it can be expected that such effects also develop over time and that students need less time overall for solving a task. The transfer between contexts can also be made visible by analyzing the corresponding eye movements on components of the graph (Susac et al., 2018; Klein et al., 2019). However, to date, there is no ET study that analyzes changes in students’ problem-solving of digitally presented graphs across two domains using pretest–posttest measurements at the beginning and end of a semester.

With regard to the additional ET data from the second measurement point, the factor “time” will be integrated into the previous models by Susac et al. (2018) and Klein et al. (2019) to analyze the following hypothesis with regard to the expected developmental effects within and across the two domains and contexts on the relationship between dwell times and test scores:

• H3: The dwell time on the tasks and the individual graph components [areas of interest (AOIs)] is lower at the second measurement point for students from both domains and in both contexts.

## Materials and Methods

### Sample

As a postreplication study, we based the present article on the sample of the replication study by Klein et al. (2019) and carried out a second measurement at the end of the winter semester 2018/19. The first measurement (t1) took place during the first weeks of the students’ first semester. The students were tested again at the end of the semester (t2). During the semester, they attended courses and learned about graphs in their respective domains. At t2, the same graph tasks were presented to the students. Study participation was voluntary and was compensated with 20€.

In total, 41 first-year students (matched sample) from the initial study of Klein et al. (2019) participated in the experiment again (Table 1): 20 physics students and 21 economics students. The average age in the sample was 20.27 years, with physics students being slightly younger (19.95 years) than economics students (20.57 years). The grade for higher education entrance qualification also differs systematically between the physics [P] and the economics students [E] [(*t* = −2.784, *p* = 0.009, *d* = 0.972); mean (SD) *P* = 1.79 (0.478); mean (SD) *E* = 2.25 (0.469)]; 89% of the physics students took an advanced physics course in upper secondary education, whereas only 16% of the economics students attended advanced courses. For an extended description of the sample, see Klein et al. (2019).

**TABLE 1:** Comparison of the postreplication study with the original study by Susac et al. (2018) and the replication study from Klein et al. (2019).

	This study	Klein et al., 2019	Susac et al., 2018
Participants	20 physics students (first year), 21 economics students at t1 and t2	27 physics students (first year), 40 economics students	45 physics students (teacher program, fourth year), 45 psychology students
Materials	Four isomorphic pairs of items about graph slope and area under a curve in the context of physics and economics (finance)		
Apparatus	Tobii X3–120 Hz		SMI RED500 Hz
Additional data	Confidence scores		Student strategies (questionnaire)
Coding scheme	Only direct response (correct or incorrect)		Response and explanation (correction)
Data analysis	ANOVAs to determine the effects of question type, concepts, group, and context on the dependent variables Area of interest (AOIs) question, graph, multiple choice, axis labels, axis tick labels		
Analytic focus	Analysis of student change between t1 and t2	Saccadic direction, attention distribution	Analysis of student strategies

### Tasks

To assess students’ graph understanding within and across domains, graphs that are regularly used in both domains and are important for learning domain-specific concepts are required. Linear graphs are used extensively in both physics (Klein et al., 2019) and economics (Benedict and Hoag, 2012) and are clearly distinguishable from other forms of graphical representation (e.g., pie charts, Venn diagrams) (Kosslyn, 1999). Although other graphs are also used in both domains, our study focuses on one single type of graph to avoid distortions caused by the graph type (Strobel et al., 2018).

**FIGURE 1:**
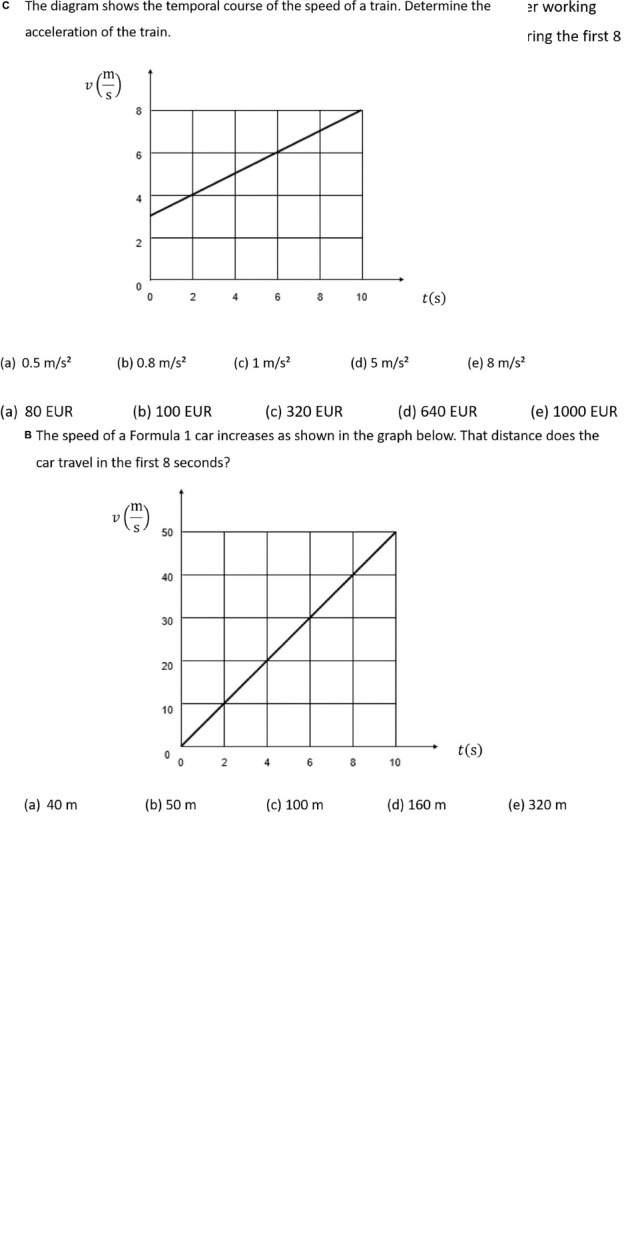
Isomorphic task examples: **(A)** quantitative “question type” and the graph concept “area” for economics “context”; **(B)** quantitative “question type” and the graph concept “area” for physics “context”; **(C)** quantitative “question type” and the graph concept “slope” for physics “context” (the “qualitative” concept slope for both contexts can be seen in Klein et al., 2019).

The study presented here used four isomorphic pairs of line graph tasks (4 from physics and 4 from economics) as they were used by Susac et al. (2018) and Klein et al. (2019) (Figure 1). A 2 × 2 × 2 (context × question × concept) design was applied, in which each task belongs either to the domain of physics or economics (context), contains either the graph concept of “slope” or “area” (concept), and requires either a mathematical calculation or purely verbal reasoning from the participants (type of question) (for an example, see Figure 1).

All tasks are presented in a closed-ended format and comprise a question of one or two sentences, a graph, and one correct and up to four incorrect response options. Each graph task also comprises one or two linear curves and other common elements like *x*-axis and *y*-axis.

### Apparatus and ET Analysis

**FIGURE 2:**
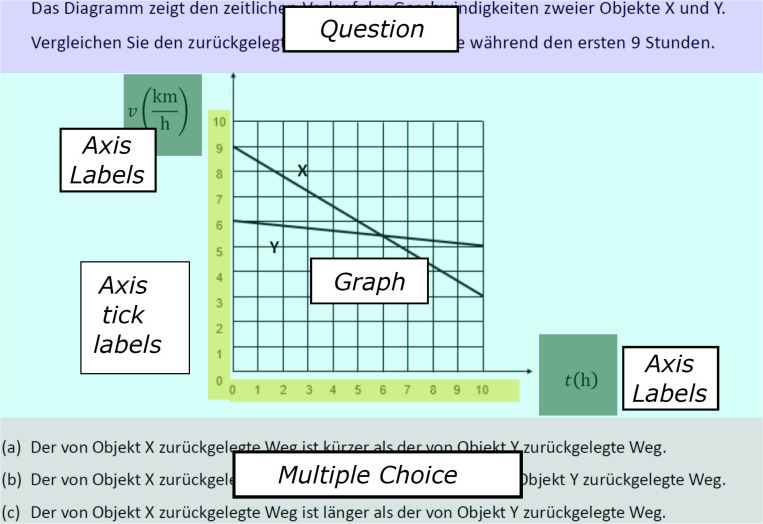
Areas of interest (AOIs) of a graph task (adapted from Klein et al., 2019, p. 6).

To perform the ET study, the graph comprehension tasks were presented to the students on a 22-inch computer screen (1,920 × 1,080 pixels). A Tobii Pro X3-120 (120 Hz), which is positioned below the monitor and is not worn by the test taker, was used to record the ET data. The visual angle resolution was below 0.4°. The dwell time (eye movements below an acceleration of 8,500°/s^2^ and a velocity below 30°/s) was assessed and used to measure the students’ focus on selected AOIs in the tasks (Figure 2). The AOIs included the task question, the graph itself, axes, and the response options.

After a 9-point calibration process, the eight tasks were presented to the students in a random order, and ensuring that two subsequent tasks were never equal in concept and type of question to avoid students realizing that some tasks only varied in context and realizing that they just need to apply the same task-solving strategy. The order in which the tasks were administered to the students also ensured that isomorphic tasks were never presented one directly after the other. After viewing a task, the test takers had to click and choose one answer from the presented response options using a mouse. Then they had to rate how confident they were that their chosen response option was correct on a six-point Likert scale ranging from very high confidence to very low confidence. By pressing the spacebar, the test takers could proceed to the next task. After the test, each task was coded with 1 if a student chose the correct response (attractor) and 0 if a student chose one of the distractors (maximum score: 8 points). The confidence rating and the task score sum were linearly transformed into a scale reaching from 0 to 100, with 0 indicating low scores and low confidence and 100 indicating high scores and high confidence.

After completion of all graph tasks (at t1 and t2), paper-and-pencil questionnaires were administered to collect sociodemographic data (e.g., gender, school education, school leaving grade; for details, see Klein et al., 2019).

### Statistical Approaches

To answer the research questions, several repeated-measures analyses of variance (ANOVAs) were performed, which were also used by Susac et al. (2018) and Klein et al. (2019). This allowed us to systematically explore the relationships between task characteristics (*context*, *concept* and type of *question*s) and examined domains (physics vs. economics) on the basis of the final test scores and to make the comparison of the findings between the three studies transparent. The *measurement point* (t1: beginning of first semester or t2: end of first semester), the *context*, the *concept*, and the *question* were modeled as within-subject factors, and the domain (physics vs. economics) as intermediate subject factor.

To test the null hypothesis that variance is equal across domains and measurement points, Levene test was used, and the assumption of homogeneity of variance was met for every ANOVA. Analogous to Susac et al. (2018) and Klein et al. (2019), correlations were calculated using the Bravais–Pearson correlation coefficient.

As with the test scores analysis, repeated-measures ANOVAs were performed to analyze the dwell times, taking into account the task characteristics and domains. In addition to the total dwell time during task processing, the dwell times on task-relevant AOIs and on the task questions were analyzed. The total processing time can vary at the second measurement because the test takers are familiar with the type of tasks, recognition effects may occur, and they have attended domain-specific classes in which they learned about graphs in their specific contexts.

## Results

### Changes in Students’ Test Scores Within and Across Domains (H1)

The mean test score of the pretest–posttest sample was (60% ± 27%) in t1 and (65% ± 25%) in t2, with a change with a small effect size (Cohen, 1988) [*t*(40) = 1.366, *p* = 0.18, *d* = 0.21]. A comparison of the two domains shows that the physics students achieved better results at both measurement points [t1: (70% ± 27%), t2: (78% ± 18%)] than the economics students [t1: (49% ± 24%), t2: (52% ± 23%)]. They also show a comparatively higher increase of about 10% in the test score than the economics students with about 6%. An ANOVA with repeated measurements (t1 and t2) as inner-subject factor and the domain (physics students and economics students) as intermediate subject factor showed that the mean test score difference between the domains is also significantly higher with a large effect size [*F*(1, 39) = 13.355; *p* = 0.001; η^2^_p_ = 0.255]. However, no significant differences in the increase from t1 to t2 between the two domains, which are mapped by the interaction term (time × domain), can be identified [*F*(1, 39) = 0.293 *p* = 0.592; η^2^_p_ = 0.007]. Thus, students from both domains showed a similar increase in the overall test score.

Next, the changes in the test score between the two measurements (t1 and t2) were examined with regard to the question type (qualitative vs. quantitative) and the concept (graph “slope” vs. “area” under the curve). A two-way repeated-measures ANOVA was conducted for each domain. For physics students, a statistically significant main effect was found only for the type of question [*F*(1, 19) = 14.968, *p* = 0.001; η^2^_p_ = 0.441] and no effect for time [*F*(1, 19) = 1.667, *p* = 0.212, η^2^_p_ = 0.081] or concept [*F*(1, 19) = 3.449, *p* = 0.079; η^2^_p_ = 0.154]. The interaction effects were not significant either. For economics students, a significant general time effect was not evident [*F*(1, 20) = 0.373, *p* = 0.548; η^2^_p_ = 0.018], but significant effects for question [*F*(1, 20) = 39.174, *p* = 0.000; η^2^_p_ = 0.662], concept [*F*(1, 20) = 21.774, *p* = 0.000; η^2^_p_ = 0.521], and the time × concept interaction [*F*(1, 20) = 14.440, *p* = 0.001; η^2^_p_ = 0.419] were found.

**FIGURE 3:**
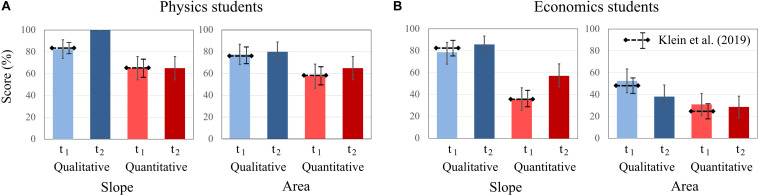
Average scores of **(A)** physics students and **(B)** economics students on the qualitative and quantitative questions about graph slope and area under a graph at t1 and t2. The error bars represent 1 standard error of the mean (SEM). The dashed lines represent the mean and error bars of the total sample of Klein et al. (2019).

Similar to Klein et al. (2019), both physics and economics students scored higher on qualitative than on quantitative tasks. Economics students generally scored higher on tasks that cover the concept of “slope” than on tasks on the concept of “area.” Furthermore, for economics students, there are differences in the changes of the test scores between the two concepts. Economics students’ scores increase on items of “slope” [t1: 57.14%; t2: 71.43%] but decrease on “area” tasks [t1: 41.66%; t2: 33.33%]. Other interaction effects were not significant. For the economics students, the difference between scores on qualitative and quantitative tasks was larger for questions about “slope” from t1 to t2 (Figure 3). “Slope” tasks with quantitative requirements show the largest increase in the scores of economics students.

For physics students, the biggest change was in the test scores of qualitative graphs on “slope” from t1 to t2. In t2, all physics students solved these items correctly.

To compare students from both domains across both contexts, we applied a repeated-measures ANOVA with time and context (physics vs. finance) as a within-subject factor and with the domain (physics vs. economics) as a between-subject factor. The analysis was performed for each pair of isomorphic tasks (Table 2). Similar to Klein et al. (2019), for qualitative tasks about “slope,” significant differences for time but no other main or interaction effects were found. For quantitative tasks on “slope,” a significant main effect was found only for task context, indicating that across both measurements (t1 and t2) and domains, students generally scored higher on tasks with a physics context than tasks with a finance context. Compared to Klein et al. (2019), students from both domains still solved physics tasks better than finance tasks, although economics students’ scores on quantitative tasks on “slope” in their own domain increased significantly [*t*(20), *p* = 0.017, *d* = 0.567] (Table 2).

**TABLE 2:** Results of the two-way ANOVAs conducted on the students’ scores with the time (t1 vs. t2) and the context (physics vs. finance) as within-subject factors and with the domain (physics students vs. economics) as a between-subject factor.

	Time			Domain			Context		
	*F*	*p*	$\eta_{\text{p}}^{2}$	*F*	*p*	$\eta_{\text{p}}^{2}$	*F*	*p*	$\eta_{\text{p}}^{2}$
“Slope” qualitative	5.075	0.030	0.115	1.839	0.183	0.045	0.384	0.539	0.010
“Slope” quantitative	2.518	0.121	0.061	3.592	0.065	0.084	4.393	0.043	0.101
“Area” qualitative	0.955	0.334	0.024	15.678	0.000	0.287	14.158	0.001	0.266
“Area” quantitative	0.133	0.717	0.003	9.030	0.005	0.188	0.556	0.460	0.014

	**Time × domain**			**Time × context**			**Time × domain × context**		
	***F***	***p***	**$\eta_{\text{p}}^{2}$**	***F***	***p***	**$\eta_{\text{p}}^{2}$**	***F***	***p***	**$\eta_{\text{p}}^{2}$**

“Slope” qualitative	0.896	0.350	0.022	0.274	0.604	0.007	0.000	0.990	0.000
“Slope” quantitative	2.518	0.121	0.061	1.448	0.236	0.036	1.448	0.236	0.036
“Area” qualitative	1.938	0.172	0.047	0.049	0.826	0.001	0.503	0.482	0.013
“Area” quantitative	0.495	0.486	0.013	0.001	0.971	0.000	2.266	0.140	0.055

	**Domain × context**								
	***F***	***p***	**$\eta_{\text{p}}^{2}$**						
						
“Slope” qualitative	0.000	0.988	0.000						
“Slope” quantitative	0.275	0.603	0.007						
“Area” qualitative	0.337	0.565	0.009						
“Area” quantitative	8.036	0.007	0.171						

*The specification F(1.39) applies to all F values of the following analyses of variance.*

**FIGURE 4:**
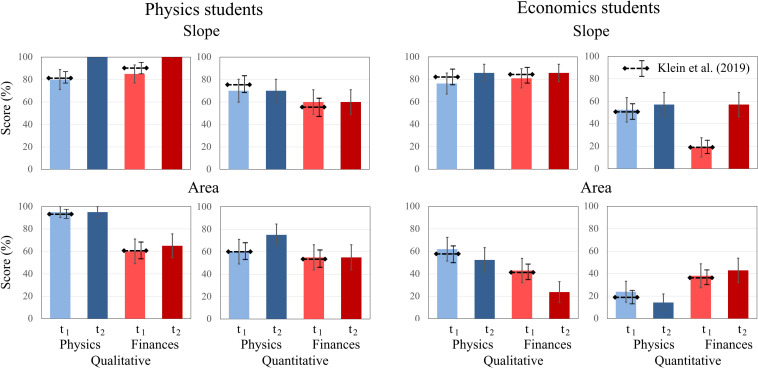
Average scores of physics and economics students in the contexts of physics and finance on the qualitative and quantitative tasks about graph slope and area under a graph at t1 and t2. The error bars represent 1 standard error of the mean (SEM). The dashed lines represent the mean and error bars of the total sample of Klein et al. (2019).

Compared to Klein et al. (2019), physics students had higher scores at both t1 and t2 on qualitative tasks on the “area under the curve” than economics students. The economics students’ scores on “area” tasks differ from their scores on all other types of task. From t1 to t2, their test scores decreased in the physics context and increased slightly in the finance context, and both for qualitative and quantitative tasks (Figure 4). Thus, significant domain effects for both tasks were found, but no time × domain × context effect occurred (Table 2).

Overall, physics students scored significantly better on physics tasks than on finance tasks at t1 [*t*(19) = 2.131, *p* = 0.046, *d* = 0.466] and t2 [*t*(19) = 3.040, *p* = 0.007, *d* = 0.68]. The effects for economics students were not significant, although they increased their score on finance tasks (t1: 45% ± 21%, t2: 52% ± 28%) more than their score on physics tasks (t1: 54% ± 30%, t2: 52% ± 25%).

### Changes in Students’ Confidence Ratings Within and Across Domains (H2)

The mean confidence rating and standard deviation were [t1: (61% ± 25%), t2: (67% ± 20%)]. The physics students showed a confidence level of [t1: (65% ± 29%), t2: (73% ± 22%)] and the economics students of [t1: (57% ± 21%), t2: (61% ± 17%)], with no significant differences between the two domains in t1 and t2 (*p* > 0.05). For the physics students, the total test score and the mean confidence level correlated highly in t1, but did not significantly correlate in t2 [t1: *r*(20) = 0.621, *p* < 0.01; t2: *r*(20) = 0.173, *p* > 0.05], whereas for the economics students, there was no significant correlation at either measurement [t1: *r*(21) = 0.198, *p* > 0.05; t2: *r*(21) = −0.297, *p* > 0.05].

**FIGURE 5:**
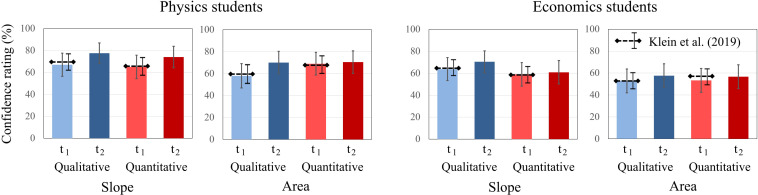
Average confidence ratings of physics students and economics students on the qualitative and quantitative tasks about graph slope and area under a graph at t1 and t2. The error bars represent 1 standard error of the mean (SEM). The dashed lines represent the mean and error bars of the total sample of Klein et al. (2019).

To further explore students’ confidence ratings, the same analysis procedure was applied as for the test scores. Two-way ANOVAs revealed no significant main effects for the factors time, concept, and type of question for physics students. However, for economics students, the factor concept was significant [*F*(1, 20) = 5.906, *p* < 0.05, η^2^_p_ = 0.228]. No significant interaction effects between time, concept, and type of question were revealed for either domain. For both question types about graph “slope,” students’ confidence ratings increased for both domains from t1 to t2, while the increase was more pronounced in physics students. The same applies to both question types about “area” graphs, even though the increase in confidence ratings was weaker for both domains compared to “slope” graphs. For both domains, confidence ratings were higher for “slope” graphs at t1 and t2 compared to “area” graphs (Figure 5).

To analyze the impact of context and time on students’ confidence ratings, a repeated-measures ANOVA was run with context and time as the within-subject factors and domain as the between-subject factor for each pair of isomorphic tasks. The results are shown in Table 3.

**TABLE 3:** Results of the two-way ANOVAs conducted on the students’ confidence ratings with the time (t1 vs. t2) and the context (physics vs. finance) as within-subject factors and with the domain (physics students vs. economics) as a between-subject factor.

	Time			Domain			Context		
	*F*	*p*	$\eta_{\text{p}}^{2}$	*F*	*p*	$\eta_{\text{p}}^{2}$	*F*	*p*	$\eta_{\text{p}}^{2}$
“Slope” qualitative	4.997	0.031	0.114	0.395	0.534	0.010	0.421	0.520	0.011
“Slope” quantitative	1.191	0.282	0.030	2.652	0.111	0.064	0.211	0.649	0.005
“Area” qualitative	4.914	0.033	0.112	1.708	0.199	0.042	1.299	0.261	0.032
“Area” quantitative	0.565	0.457	0.014	3.399	0.073	0.080	0.707	0.406	0.018

	**Time × domain**			**Time × context**			**Time × domain × context**		
	***F***	***p***	**$\eta_{\text{p}}^{2}$**	***F***	***p***	**$\eta_{\text{p}}^{2}$**	***F***	***p***	**$\eta_{\text{p}}^{2}$**

“Slope” qualitative	0.249	0.620	0.006	0.019	0.890	0.000	1.062	0.309	0.027
“Slope” quantitative	0.504	0.482	0.013	1.217	0.277	0.030	0.754	0.390	0.019
“Area” qualitative	0.916	0.344	0.023	3.240	0.080	0.077	12.378	0.001	0.241
“Area” quantitative	0.081	0.777	0.002	1.083	0.305	0.027	0.136	0.715	0.003

	**Domain × context**								
	***F***	***p***	**$\eta_{\text{p}}^{2}$**						
						
“Slope” qualitative	0.714	0.403	0.018						
“Slope” quantitative	1.675	0.203	0.041						
“Area” qualitative	3.046	0.089	0.072						
“Area” quantitative	0.014	0.905	0.000						

*The specification F(1.39) applies to all F values of the following analyses of variance.*

For qualitative tasks of “slope” and “area” under a curve, significant main effects for time were found. The students’ confidence increased from t1 to t2 for all qualitative tasks, but not for quantitative tasks. Furthermore, a significant time × domain × context effect was identified for qualitative tasks on “area” under a curve, showing that physics students’ confidence increased over time for each context, whereas economics students’ confidence increased over time for finance tasks and decreased over time for physics tasks. All other main and interaction effects were not significant (Table 3).

**FIGURE 6:**
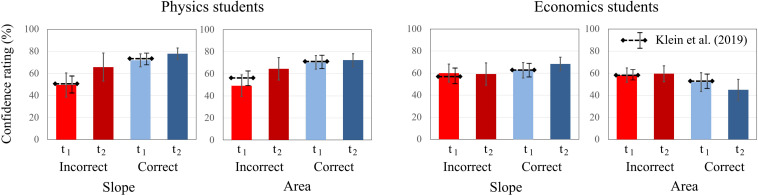
Average confidence ratings of physics and economics students related to correct and incorrect responses on the slope tasks and on the area tasks at t1 and t2. The error bars represent 1 standard error of the mean (SEM). The dashed lines represent the mean and error bars of the total sample of Klein et al. (2019).

To investigate the accuracy of students’ confidence, the ratings for correct and incorrect responses at each measurement (t1 and t2) were considered. Because of this split of the data across measurements and test scores, and the lack of paired variables (there is only one confidence rating for either a correct or an incorrect response), a repeated-measures analysis was not possible. Hence, all tasks on the “slope” concept and on the “area” concept were aggregated, respectively (Figure 6). For the “slope” concept, the physics students were significantly more confident when responding correctly than when responding incorrectly at t1 but not at t2 [t1: *t*(78) = 2.708, *p* = 0.008; t2: *t*(78) = 1.559, *p* = 0.123]. In contrast, the economics students’ confidence was not significantly different between correct and incorrect responses [t1: *t*(82) = 0.362, *p* = 0.718; t2: *t*(82) = 1.369, *p* = 0.175]. For the “area” concept, the results were similar for physics students; whereas they responded correctly with higher confidence at t1, there was no significant difference at t2 [t1: *t*(78) = 2.915, *p* = 0.005; t2: *t*(78) = 1.174, *p* = 0.275]. For economics students, the results were different (Figure 6): At t1, they reported a higher confidence in their incorrect responses than their correct responses, although this difference was not significant. At t2, this difference increased, indicating that their confidence was significantly higher for incorrect responses than for correct responses [t2: *t*(82) = −2.810, *p* = 0.006]. Although there is an increase of overall confidence at t2, the self-assessment of students in both domains was less accurate at t2 than at t1.

### Changes in Students’ Dwell Times Within and Across Domains (H3)

#### Total Dwell Time

The analysis of students’ eye movements is based on their total dwell time on the tasks before responding and then rating their confidence. The physics students had an average total dwell time of 412 ± 86 s at t1 and 333 ± 75 s at t2. The economics students needed 461 ± 172 s at t1 and 346 ± 125 s at t2 to respond to all tasks.

**FIGURE 7:**
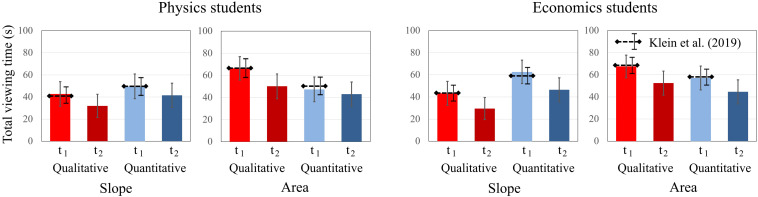
Average total dwell time of physics students and economics students on the qualitative and quantitative tasks about graph slope and area under a graph at t1 and t2. The error bars represent 1 standard error of the mean (SEM). The dashed lines represent the mean and error bars of the total sample of Klein et al. (2019).

To compare students’ total dwell time on qualitative and quantitative tasks about graph “slope” and the “area” under a curve, an ANOVA was conducted separately for both domains including time, question, and concept as between factors. For physics students, significant main effects for time [*F*(1, 19) = 13.838; *p* = 0.001; η^2^_p_ = 0.421] and concept [*F*(1, 19) = 11.291; *p* = 0.003; η^2^_p_ = 0.373] were found. The factor question type was not significant, but there was a significant interaction effect for question × concept [*F*(1, 19) = 11.244; *p* = 0.003; η^2^_p_ = 0.372]. Physics students spent less time on tasks at t2 and spent more time viewing the “area” tasks than the “slope” tasks (Figure 7). The significant interaction effect is similar to Klein et al. (2019), indicating that the question had the opposite effect. Physics students paid more attention to quantitative “slope” tasks and to qualitative “area” tasks.

The effects were similar for economics students. While there were significant main effects for time [*F*(1, 20) = 13.257; *p* = 0.001; η^2^_p_ = 0.436] and concept [*F*(1, 20) = 9.199; *p* = 0.007; η^2^_p_ = 0.315], the factor question type was not significant. There was a significant interaction effect for question × concept [*F*(1, 20) = 13.257; *p* = 0.002; η^2^_p_ = 0.399]. Economics students also spent less time on tasks at t2 and spent more time viewing the “area” tasks than the “slope” tasks. The significant interaction effect also persists for the economics students. Overall, we found no significant differences between the students’ dwell times at t1 and t2.

To further explore students’ total dwell times, the same analysis was applied as for the test scores and the confidence ratings. The results of a two-way mixed-design ANOVA with the between-subject factor domain and the within-subject factor context for each pair of isomorphic tasks are shown in Table 4. The analysis revealed no main effect of context or domain. The interaction domain × concept, however, was significant, indicating that physics students needed less time to respond to tasks from the physics context, whereas economics students needed less time to respond to tasks from the finance context. This finding is similar to Klein et al. (2019).

**TABLE 4:** Results of the two-way ANOVAs conducted on the students’ dwell times with the time (t1 vs. t2) and the context (physics vs. finance) as within-subject factors and with the domain (physics students vs. economics) as a between-subject factor.

	Time			Domain			Context		
	*F*	*p*	$\eta_{\text{p}}^{2}$	*F*	*p*	$\eta_{\text{p}}^{2}$	*F*	*p*	$\eta_{\text{p}}^{2}$
“Slope” qualitative	22.951	0.000	0.370	0.083	0.775	0.002	0.914	0.345	0.023
“Slope” quantitative	8.626	0.006	0.181	1.203	0.279	0.030	1.752	0.193	0.043
“Area” qualitative	17.616	0.000	0.311	0.128	0.723	0.003	0.163	0.688	0.004
“Area” quantitative	3.414	0.072	0.080	0.858	0.360	0.022	0.080	0.779	0.002

	**Time × domain**			**Time × context**			**Time × domain × context**		
	***F***	***p***	**$\eta_{\text{p}}^{2}$**	***F***	***p***	**$\eta_{\text{p}}^{2}$**	***F***	***p***	**$\eta_{\text{p}}^{2}$**

“Slope” qualitative	0.343	0.562	0.009	0.009	0.925	0.000	9.126	0.004	0.190
“Slope” quantitative	0.954	0.335	0.024	0.302	0.586	0.008	0.566	0.457	0.014
“Area” qualitative	0.027	0.871	0.001	4.301	0.045	0.099	0.316	0.577	0.008
“Area” quantitative	0.797	0.377	0.020	3.217	0.081	0.076	0.856	0.361	0.021

	**Domain × context**								
	***F***	***p***	**$\eta_{\text{p}}^{2}$**						
						
“Slope” qualitative	5.614	0.023	0.126						
“Slope” quantitative	2.102	0.155	0.051						
“Area” qualitative	0.958	0.334	0.024						
“Area” quantitative	0.556	0.460	0.014						

*The specification F(1.39) applies to all F-values of the following analyses of variance.*

Regarding differences of total dwell time between t1 and t2, significant differences were found for almost each pair of isomorphic tasks. Only the total dwell time in quantitative “area” tasks was slightly below the level of significance (*p* > 0.05). Overall, students needed less time at t2, but as the effect sizes indicate, there were fewer differences between the two measurements for quantitative tasks.

#### Dwell Time on Different Areas of Interest (AOI)

**FIGURE 8:**
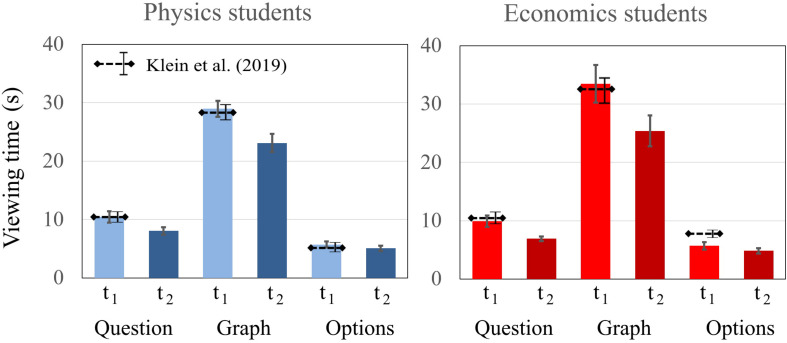
Average fixation time of physics and economics students on the AOIs question, graph, and multiple choice at t1 and t2. The error bars represent 1 standard error of the mean (SEM). The dashed lines represent the mean and error bars of the total sample of Klein et al. (2019).

In the sample of Klein et al. (2019), no differences were found between physics students and economics students in the defined AOIs (question, graph, and multiple choice) at t1. Compared to Klein et al. (2019), the findings presented here did not differ significantly. Students’ dwell times on the AOIs (question, graph, and multiple choice) were compared between the domains. Six Bonferroni-adjusted *t* tests showed no statistical difference between the dwell time of physics and economics students on the AOIs question [t1: *t*(39) = 0.388, *p* = 0.700; t2: *t*(39) = 1.530, *p* = 0.134], graph [t1: *t*(39) = −1.262, *p* = 0.214; t2: *t*(39) = −0.723, *p* = 0.474], and multiple choice [t1: *t*(39) = 0.012, *p* = 0.990; t2: *t*(39) = 0.321 *p* = 0.750]. There was a similar drop of total dwell time from t1 to t2 for students from both domains. In the comparison of the two measurements, there were also significant differences in the AOIs of question and graph between t1 and t2. However, no significant differences for the AOI multiple choice between t1 and t2 were found for either physics or economics students (Figure 8).

Next, the total dwell time on the AOI axis labels (adding the dwell times on the *x*-axis and *y*-axis labels) was determined for each item. A two-way mixed-design ANOVA with the between-subject factor domain and the within-subject factors time and context on total dwell time on the AOI axis labels was performed, indicating a significant main effect of time [*F*(1, 39) = 18.196; *p* < 0.001; η^2^_p_ = 0.318]. In contrast to Klein et al. (2019) and Susac et al. (2018), no interaction effects were found, even when considering the effects only at t2 (*p* > 0.05). Because of the drop of total dwell times, dwell times on axis labels were also not significantly different between students from the two domains.

**FIGURE 9:**
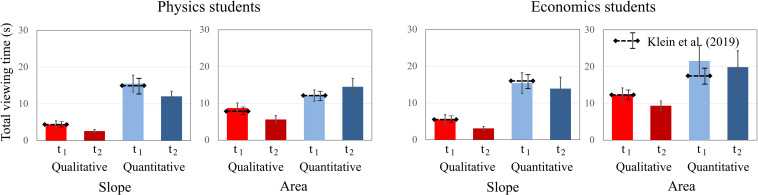
Average total dwell time of physics students and economics students on the AOI axis tick labels for qualitative and quantitative questions about graph slope and area under a graph at t1 and t2. The error bars represent 1 standard error of the mean (SEM). The dashed lines represent the mean and error bars of the total sample of Klein et al. (2019).

The dwell times on the axis tick labels were analyzed by a mixed-design ANOVA including time, question, and concept as within-factors for each domain. There was a significant main effect of question type [*F*(1, 19) = 39.752; *p* < 0.001; η^2^_p_ = 0.677] and a significant interaction effect of time × question × concept [*F*(1, 19) = 4.891; *p* = 0.039; η^2^_p_ = 0.205] for physics students. Other effects were not significant. Similar to Klein et al. (2019), physics students paid more attention to the axes when responding to quantitative than to qualitative tasks and especially paid more attention to the axes of quantitative “area” tasks at t2 in contrast to the quantitative “slope” tasks (Figure 9). For the economics students, the main effects of the factors concept [*F*(1, 20) = 11.491; *p* = 0.003; η^2^_p_ = 0.365] and question type were significant [*F*(1, 20) = 19.976; *p* < 0.001; η^2^_p_ = 0.500], but the effect for the factor *time* was not. The interaction effects were not significant. Economics students also paid more attention to the axis tick labels of quantitative tasks and to the axis tick labels of tasks about the “area under the curve.” All findings were similar to Klein et al. (2019).

## Discussion

### Changes in Scores Across Contexts and Domains

H1: Physics and economics students solve graph tasks related to the subject they are enrolled in more successfully at the second measurement point than at the first measurement point.

However, the findings indicate differences in the development of graph understanding that are related to the task *context* and the task *concept*. Similar to the results of Klein et al. (2019), the physics students outperformed the economics students in terms of overall test performance and, in particular, achieved higher scores on tasks from the physics context at both t1 and t2. On average, at t2, the physics students also performed better on finance tasks than the economics students. In particular, they achieved higher scores on qualitative tasks on the concept of “slope” in the finance context at t2 than the economics students. On “area” tasks in finance, the scores of physics students remained at a similar level, and the scores of economics students increased from t1 to t2, whereas in the physics context, the scores of economics students decreased. These findings indicate that the physics students were more successful in transferring the graph task solution strategies that they had consolidated over the semester to other task contexts.

For the economics students, an increased “transfer effect” of this kind can be seen for the “slope” tasks, as the economics students achieved higher scores on the qualitative and quantitative tasks in the physics context at t2 than at t1. Overall, however, their scores at t2 (and t1) are lower than those of the physics students. The economics students’ scores on “area” tasks in the physics context were lower at t2 than at t1, whereas the physics students achieved higher scores as well as greater graph understanding gains. Whereas at t1 the economics students achieved higher scores in only one of four task pairs in the finance context (Klein et al., 2019), at t2 the opposite became evident for the subsample considered here. Even when taking into account the declining scores in the quantitative “area” tasks in the physics context, at t2 the economics students achieved higher scores in three of four task pairs in the finance context. This indicates that students experience context-specific learning effects that become evident when they expand or transfer their graph solving skills to another context. Similar findings have been reported in previous research, where the transfer of graph understanding over a period of time was different for students from different domains, and the concepts the students were required to use to solve the tasks also differed (Jerry and Aaron, 2010; Bergey et al., 2015; Miller et al., 2016).

Similar to the results of both reference studies (Susac et al., 2018; Klein et al., 2019), at t2, “area” tasks were solved less successfully than “slope” tasks by students from both domains (physics and economics). The qualitative tasks related to the concept of “slope” were solved more successfully across both domains at t2 than at t1 (80%; Klein et al., 2019), with a correct solution rate of about 92%. The very high solution rate at t2 is in line with the results of our curriculum analyses, because tasks of this kind are an integral part of the curriculum in both domains. “Retest effects” are less likely to occur as the students were not given the solutions to the tasks and more than 3 months had passed between t1 and t2.

For the economics students, an increase in their scores on “slope” tasks from the physics context was also determined at t2, indicating a similar understanding of the representation of this concept in physics and finance graphs. Furthermore, there is a high increase in scores on the quantitative “slope” tasks in the finance context. Fundamental mathematical concepts are taught in economics degree programs right at the beginning of the curriculum, which enables students to understand and analyze subject-related phenomena using these methodological tools (Jensen, 2011; Benedict and Hoag, 2012). Teaching in the domain of economics in particular places a strong focus on the concept of “slope” (e.g., in the analysis of extremes, cost, and profit trends), which is also generally easier for students to comprehend than the concept of “area under the curve.” Similar findings were reported for first-year students from standardized assessments in higher economics education, which also include tasks that refer to the “slope” concept (e.g., the Test of Understanding in College Economics, Walstad et al., 2007; or the German WiWiKom-Test, Zlatkin-Troitschanskaia et al., 2019), but not to the concept of “area under the curve.”

Differences in scores also occur with respect to the type of question. Both at t1 in the overall sample (Klein et al., 2019) and at t1 and t2 in the subsample examined in this study, qualitative tasks were always solved more successfully than quantitative tasks. Solving a mathematical task appears to require more cognitive resources – which may be measured, for instance, by assessing cognitive load (Gegenfurtner et al., 2011) – than solving a graph task with purely textual requirements (for similar findings, see, e.g., Curcio, 1987; Woolnough, 2000; Benedict and Hoag, 2012; Laging and Voßkamp, 2017; Ceuppens et al., 2019; Shavelson et al., 2019). This finding is also in line with research on mathematical requirements in graph tasks (Curcio, 1987; Planinic et al., 2013; Susac et al., 2018; Wemyss and van Kampen, 2013).

Apart from the “area” tasks, the number of students who had already scored high in the qualitative “slope” tasks at t1 further increased their scores at t2. In the qualitative “area” tasks, higher scores were also identified at t2 for both domains, whereas only physics students succeeded in increasing their scores in the quantitative “area” tasks in the physics context. The finding that the highest score increases or the greatest score decreases in the respective familiar contexts (i.e., “area”/finance and “slope”/finance for economics students; “area”/physics for physics students) occur in the quantitative tasks illustrates that the difficulties the students had with these tasks at the beginning of the semester remained at t2. One possible explanation is that fundamental mathematics courses, which also include graph understanding, are taught in both courses (physics and economics) primarily in the first semesters (for physics, see Küchemann et al., 2019; for economics, see Jensen, 2011).

In summary, despite the discussed differences in terms of *domains*, task *contexts*, task *concepts*, and the *type of question*, H1 cannot be rejected, but more comprehensive research on the graph understanding of students in different domains and contexts is urgently needed.

### Change in Confidence Across Contexts and Domains

H2: Physics and economics students rate their confidence in their solution to tasks related to the subject they are enrolled in more accurately at the second measurement point than at the first measurement point.

A comparison of the two measurements shows that at t2, the confidence rating has only slightly, but not significantly, increased. When looking at the *context*, we did not find any statistically significant effects. Moreover, similar to Klein et al. (2019), there is no significant difference between the students from both *domains*. With regard to the task *concept*, apart from the “area” task solutions of the economics students, the confidence rating of correct solutions increased at t2. With the exception of economics students’ solutions to the “slope” tasks, incorrect solutions were self-assessed as being correct with more confidence at t2. Already at t1 (Klein et al., 2019), approximately 50% of incorrect solutions were self-assessed as correct, indicating the students’ deficient metacognitive skills. This effect increased to greater than 60% at t2. This finding is also in line with numerous studies across disciplines (Nowell and Alston, 2007; Bell and Volckman, 2011; Guest and Riegler, 2017; Brückner and Zlatkin-Troitschanskaia, 2018).

The increasing confidence in one’s own erroneous solving strategies for graph tasks can be traced back to causes described under the umbrella term “error knowledge,” which includes, for instance, overestimating one’s (task-related) knowledge and skills and deficits in the ability to diagnose errors in the solution process (Kruger and Dunning, 1999). In particular, the latter one can also be caused by didactic priorities. Generally, students are taught to identify possible strategies that will lead them to correct solutions. However, they are less systematically taught to recognize systematic errors in their solution process.

The negative change in the self-assessment of economics students on “area” tasks is particularly remarkable. Compared to all other tasks, both the average correct solution rate and the average correct self-assessment for these tasks decrease significantly. Apparently, there is no recognition effect but a learning effect, so that even wrong task solutions were self-assessed as correct remarkably often. For qualitative tasks on “area under a curve,” physics students’ confidence increased over time for each task context, whereas economics students’ confidence increased for economics tasks and decreased for physics tasks.

In summary, students rated their correctness of responses less accurately at t2. These unexpected findings (e.g., economics students solve “area” tasks less successfully and also rate their solution less accurately at t2) indicate that the students may have developed fundamental misconceptions, which require more in-depth research in further studies. Thus, H2 cannot be confirmed, although there is an increase in confidence rating from t1 to t2 that reflects earlier findings (e.g., Guest and Riegler, 2017).

### Change in Students’ Dwell Times

H3: The dwell time on the tasks and the individual graph components (AOIs) is lower at the second measurement point for students from both domains and in both contexts.

With regard to H3, not only did the total dwell time during task processing decrease significantly at t2, but the students also spent less time reading the tasks. This may be due to a recognition effect or a learning effect in graph understanding, as the scores increased at t2, but familiarity with the tasks increased only slightly. Despite a decrease in total dwell time at t2, students from both domains still spent more time on questions about the “area under the curve” than on questions about graph “slope.” In view of the decreasing or unchanged scores (with the exception of the “area” quantitative task pair in the physics context), this further indicates the higher cognitive load that “area” tasks elicit in students (Gegenfurtner et al., 2011; Klein et al., 2019).

Furthermore, findings at t2 confirmed the findings at t1 (Klein et al., 2019) that the students’ dwell time is also longer for quantitative tasks than for qualitative tasks if the task is an “area” task. Students from both domains need longer for quantitative “area” tasks than for quantitative “slope” tasks. Thus, in line with Susac et al. (2018) and Klein et al. (2019), students need longer for complex mathematical calculations, such as “area” calculations, than for linear “slope” calculations. Longer dwell times on quantitative tasks can also be attributed to a large extent to the comparably longer dwell time on the axes. While the dwell time on the axes generally decreased at t2, it actually increased for quantitative “area” task processing by physics students. This supports the conclusion that quantitative tasks are more difficult, because comparatively more information has to be extracted from the axes and mentally processed.

Similar to Klein et al. (2019), at t2, students spent the longest time on qualitative “area” tasks. This persistent finding, which is also consistent with the decrease in processing times for all tasks, indicates that estimating the “area” is still more cognitively demanding than determining a “slope,” despite corresponding increases in knowledge. However, the effect of the novelty of such a task was not found. For qualitative tasks, students at t2 from both domains still spent more time on the axis tick labels for the “area” task compared to the “slope” task. It can be assumed that they were looking for further information to estimate the “area” size on the axes. In contrast to Klein et al. (2019), however, no longer dwell times on unfamiliar task contexts were determined at t2 compared to t1. In line with the discussed findings on transferring graph understanding between contexts, this may be due to the fact that by learning how to solve graph problems, students no longer try to decipher the meaning of the axis designations and instead have developed schemes (i.e., heuristics) that enable them to transfer the graph solution strategies from one context to another. Regarding the solving strategies, economics students still needed more time to explore the axis tick labels of qualitative “area” tasks, although they are irrelevant for the solution process. This supports the assumption that economics students use compensatory strategies to respond to these tasks. This is in line with the argument of Beichner (1994) that area estimation stimulates students’ inappropriate use of axis values.

As in Klein et al. (2019) at t1, no overall, differences in dwell times between physics and economics students were found at t2 with regard to the relationship between total dwell time and students’ performance. Intratemporal and intertemporal comparisons between both domains (physics and economics) were conducted over the three areas defined as AOIs (question, graph, multiple choice), indicating no significant differences, as students from both domains spent almost an identical amount of time on the three AOIs at t1 and t2. The significant differences in the students’ scores cannot be explained by a domain-specific change in the time spent on the graph tasks, even for t2. Thus, total dwell time alone does not explain the difference in the performance outcomes between the students from both domains.

However, a comparison of t1 and t2 shows that the time spent on the question and graph decreases significantly, but the time spent on the response options remains almost the same. This indicates that, although the students apparently extract information from the tasks more quickly, they do not have any recognition effects with regard to the task solutions, as they must look at the responses systematically again.

In summary, the effects reported here for the (sub)sample at t2 are not significantly different from those of the entire sample in Klein et al. (2019) at t1. Regarding the drop in total dwell time at t2 and the lower dwell times reported by Susac et al. (2018), there was a correlation between study progress and dwell time and the task *concept* or *question type* across both *domains*. In our study, we thus replicated the findings of both Susac et al. (2018) and Klein et al. (2019) and determined the stability of the time effect, as we found similar effects for t1 and t2. The differences between Susac et al. (2018) and Klein et al. (2019) also persist at t2; there were no significant main effects of *context* at t2. This indicates the stability of the findings over time. Thus, *H3* can be confirmed.

## Conclusion

### Summary and Future Perspectives

In a postreplication study, based on the two existing studies of Susac et al. (2018) and Klein et al. (2019), using a pretest–posttest measurement, we expanded the analytical research focus to gain initial insights about changes in students’ graph comprehension within and across domains with regard to the theoretically expected (i) time effects (measurements t1 and t2); (ii) domain effects (physics and economics); (iii) question type, concept, and context effects; and possible (vi) interaction effects.

Effects of these kinds could be found at both measurement points. For instance, physics students achieved higher scores than economics students, whereas economics students, at t2 in particular, achieved better results in tasks with a finance *context* than in physics tasks. On average, students from both domains were more likely to correctly solve tasks on the concept “slope” at both measurement points, whereas physics students correctly solved “area” tasks at 67% and “slope” tasks at 75%, and economics students correctly solved “area” tasks at 42% and “slope” tasks at 69%. Furthermore, “slope” tasks were visually processed more quickly than “area” tasks.

Overall, the accuracy of the students’ self-assessment decreased at t2, showing that overestimating incorrect solutions occurs more often than underestimating correct solutions. Further studies are needed with a particular focus on explanations for overestimating incorrect solutions and uncovering possible misconceptions, to form a basis for modified instructional research designs. For example, typical misconceptions could be discussed in classes or short interventions; for instance, digital classroom response systems can be used in larger lectures to gather data on students’ knowledge about a task concept or a solution process. This is especially important because students are increasingly using digital media to construct graphs. However, traditional media are still used in most forms of higher education instruction. Because the present study focuses only on the understanding and interpretation of graphs and not on haptic construction performance, this problem takes a backseat in the scope of this article (*Limitations and Implications*). However, empirical evidence to examine the difference between paper-based and digital understanding of graphs is also still missing. Meta-analyses would also be desirable for a consolidation of the current findings.

Similar to both referenced studies and at t2 in our study, the total dwell times and the dwell times on the defined AOIs (question, graph, and multiple choice) can hardly predict differences in the scores between the students from both domains. Instead, better predictions can be made by analyzing individual parts of the graph (e.g., axis tick labels). However, in view of the generally faster processing time and on average higher number of correct solutions, more efficient solution strategies and information processing can be assumed for students from both domains at t2. This indicated increase in the efficiency of information processing also shows that, for example, students are less irritated by the axes’ labels of unfamiliar domains.

In conclusion, the findings of the postreplication study are mostly consistent with those of the two previous studies. Subsequent studies should now be applied more specifically to the cognitive processes both within and across domains, for instance by expanding the samples and domains to investigate whether existing developments can also be found in other domains.

A more systematic exploration of graph task-relevant aspects could be conducted through expert ratings and additional ET studies with experts to investigate their solving strategies of graph tasks. Combinations of ET with other techniques, for instance, electroencephalography or skin conductance, and in particular verbal data (Leighton et al., 2017) could provide important information, for example, on the extent to which the dwell time on a certain area or the scattering of fixations is more important for solving a graph task or for maintaining existing or transferring solving strategies and emotions during learning in a domain. First insights have been provided by Susac et al. (2018), who retrospectively recorded students’ task solving strategies. Computing methods can also provide further evidence as to the extent to which eye movements are linked to information processing (Elling et al., 2012).

Further research should also focus on the consistent decrease in economics students’ scores for “area” tasks from foreign contexts, as well as on the high increase in scores for quantitative “slope” tasks at t2, while also controlling for effects of explicit instructional measures in classes, as well as possible learning opportunities outside of university. Considering that the eight isomorphic tasks are tasks that are typically used in textbooks in print or online formats, in lectures, exercises, or in (online) assessments, a more comprehensive analysis of the learning opportunities students have during the semester is required. Studies have shown, for instance, that students increasingly use digital media for their examination preparation (e.g., Wikipedia, see Maurer et al., 2020). In particular, dynamic representations of graphs or the use of graph creation software could promote graph understanding (Stern et al., 2003; Gustafsson and Ryve, 2016; Opfermann et al., 2017). For example, the dynamic hatching of an “area under a curve” with a parallel indication of the calculated values and a formula display could facilitate understanding in the sense of “learning by examples” (Schalk et al., 2020). However, the extent to which this can have a positive effect on the understanding of certain concepts like “slope” and “area” still needs to be investigated. Further research should also investigate other indicators, such as click rates (Buil et al., 2016; Hunsu et al., 2016) or the use of multiple learning media (Ainsworth, 2006; Mayer, 2009) in the context of experimental studies to provide more precise analyses of information processing and the development of graph comprehension. How students’ graph understanding and the identified differences and effects develop over the course of the degree course until their graduation should also be further investigated.

## Limitations and Implications

Despite findings that are stable over time and also in line with previous research, these results should be critically discussed in view of the limitations of this study. These limitations concern (i) the construct and the study design, (ii) the sampling, and (iii) the scope of analyses carried out so far.

(i) The study used graph tasks for two types of concepts with linear progressions, “area” and “slope,” thus capturing students’ understanding only of certain types of graphs (Curcio, 1987). Moreover, the study focus is on the students’ internal mental processes rather than on the active construction or drawing of graphs or on communication with third parties. Thus, the focus is on the recognition of trends and areas, i.e., coherent parts of a graph. Graph understanding in terms of the reproduction of individual values, interpolations between graph parts, the extrapolation and prediction of graph progressions, or interpretation in larger contexts (e.g., how an increase in inflation in 5 years will affect the economy) was not captured in this study (Curcio, 1987). Investigating such phenomena requires further task constructions and other study designs.

Furthermore, the test instrument used in this study was limited to eight tasks, which were taken as replications from previous studies; for the same reason, a treatment-control group design (e.g., in which students work on certain tasks or attend selected lectures and courses) was intentionally not used. However, because the findings and the expectations covered by the three studies have for the most part have been confirmed several times, follow-up studies, for instance, in the context of multimedia learning environments, can now be immediately conducted, at least in the investigated domains. Moreover, the present findings are primarily related to digital representations on computer screens. The extent to which extrapolation of other representational formats is possible must also be investigated in further studies.

(ii) Overall, less than two-thirds of the total sample from Klein et al. (2019) could be retested in the study at t2. Nonetheless, compared to other existing ET studies, more than 40 study participants at two measurement points constitute a considerable sample. For future studies, however, larger sample is required to generate a higher generalizability (e.g., investigating correlations of eye movements and scores in different populations), as well as an expansion at the institutional level, to include more universities, faculties, and students, for instance, to analyze teaching effects. The multilevel structure in which response behavior can vary between and within domains, previous knowledge, and other sociobiographical characteristics should be considered in an expanded sample (Hox et al., 2018). Many non-significant results in the present study can also be traced back to the sample size, so that the differences and correlations were mostly investigated with regard to effect size (see iii).

The calculation of several ANOVAs for the examined factors of graph understanding (task characteristics (context, concept, and type of questions), and domains (physics vs. economics) builds on the studies of Susac et al. (2018) and Klein et al. (2019) and extends the existing analyses by the time factor (t1 vs. t2). Because no comparable findings on the development of graph understanding are available yet, and the comparability between the studies should be ensured, an equally possible comprehensive repeated-measures ANOVA with all within-factors and the domain as between-factor specified in the study was calculated but not presented in this article. The findings of these analyses, taking into account possible inflations of standard error, lead to the same interpretations due to the significance and effect sizes.

In view of low-stakes assessments, deviations in test-taking motivation for larger samples should also be considered in appropriate empirical modeling (Penk et al., 2014). In the present study, this was only possible by means of response time effect modeling (Wise and Kong, 2005). To mitigate the potential supporting effects of test motivation, for instance, in terms of very short and very long dwell times, the study participants were offered monetary compensation, and additional individual surveys were conducted, so that negative hidden mass-group effects on test motivation were as similar as possible across all test takers.

(iii) The present study only regarded dwell times. Qualitative, retrospective interviews (Susac et al., 2018) have shown, however, that students’ task-solving strategies differ, and research could be complemented by analyses of saccadic (Klein et al., 2019) or transitional studies of fixation sequences. For example, it is conceivable that students will not only solve “area” tasks better if they look at the graph or the axis tick labels for a longer time, but also if they perform more saccadic eye movements between axis tick labels and graph. Such phenomena could be analyzed more precisely, for instance, by using process and path models.

The “area under the graph” is identified as a crucial concept in graph understanding and its development and should also be researched more intensively, especially among economics students, and treated in a differentiated manner with regard to instructional research designs. Apparently, the two types of question and the two task concept types are based on different cognitive processes, which are also addressed differently over the semester and thus lead to the changes identified in this study. Potential explanatory factors such as domain-specific prior knowledge might have an effect on these processes (e.g., students with more prior knowledge may use more efficient test-taking strategies) and should also be included in further studies. This can be done, for example, in multilevel linear mixed-effects models (Brückner and Pellegrino, 2016; Strobel et al., 2018), which take into account the structure between subject characteristics, item characteristics and response processes, and the final test scores.

## Data Availability Statement

The raw data supporting the conclusions of this article will be made available by the authors, without undue reservation.

## Ethics Statement

Ethical review and approval was not required for the study on human participants in accordance with the local legislation and institutional requirements. The participants provided their written informed consent to participate in this study.

## Author Contributions

SB wrote the manuscript and conducted the analyses. OZ-T co-wrote the manuscript and coordinated the analyses. SK, PK, and JK reviewed, corrected, and discussed the article and the analyses, and also introduced the physics perspective into the manuscript. All authors contributed to the article and approved the submitted version.

## Conflict of Interest

The authors declare that the research was conducted in the absence of any commercial or financial relationships that could be construed as a potential conflict of interest.

## Footnotes

1 In biology, for example, it is important to make developments (e.g., cell division) visible (Bergey et al., 2015; Kragten et al., 2015); in mathematics and statistics, relationships between variables, their distributions and progressions can be graphically visualized (Lichti and Roth, 2019).

2 In economics, graphs are systematically used in learning situations to explain complex phenomena (e.g., economic developments, inflation, gradients) and also in (non)standardized examinations. Graphs are also an integral part of methodological lessons focusing on modeling economic content and its graphical representation, especially in the first semesters of an economics degree course (e.g., Jensen, 2011).
